# Anti-microbial and anti-cancer efficacy of acetone extract of *Rosa chinensis* against resistant strain and lung cancer cell line

**DOI:** 10.1186/s12906-023-04222-2

**Published:** 2023-11-10

**Authors:** Kalaivani Madhavaram Kuppusamy, Sivakumar Selvaraj, Pujithaa Singaravelu, Cordelia Mano John, Kalaiselvan Racheal, Keziaann Varghese, Dinesh Kaliyamoorthy, Elumalai Perumal, Krishnamoorthy Gunasekaran

**Affiliations:** 1 Research Centre for Cellular Genomics and Cancer Research, Sree Balaji Medical College and Hospital, Chennai, 600044 India; 2 Molecular Biology section, Consultant Molecular Biologist, Medall Healthcare Private Limited, Chennai, India; 3 Department of Biomedical Sciences, Sri Ramachandra Institute of Higher Education and Research, Chennai, India; 4 Department of Biotechnology, Dr. M.G.R. Educational and Research Institute, Chennai, India; 5 Department of Microbiology, Sree Balaji Medical College and Hospital, Chennai, 600044 India; 6 Centre for Global Health Research, Saveetha Medical College, Saveetha Institute of Medical and Technical Sciences, Chennai, Tamil Nadu India; 7 Department of Medical Biochemistry, College of Health Sciences, Dambi Dollo University, KelamWelega Zone, Dembidolo, P.O. Box: 360, Oromia Region Ethiopia

**Keywords:** *Rosa chinensis*, In-vitro anti-bacterial activity, Anti-proliferative activity, Biofilm inhibition

## Abstract

**Background:**

Screening of herbal plants for various therapeutic properties is the hour as it shows promising activity. Scientific evidence of the pharmacological activity of the plant strengthens the traditional application of plants.

**Methods:**

Rose flowers (*Rosa chinensis)* were procured and grounded into a coarse powder. The DNA was isolated from rose flower and molecular identification was performed by rbcL-BF and rbcL-724R primers. Antibacterial activity was evaluated by using disc and agar diffusion methods and the anti-cancer effect of the rose flower extract (RE) was examined using MTT assay in lung cancer cell line. The mechanism of cell death induced by RE was qualitatively measured using Acridine orange/Ethidium bromide staining and Hoechst staining. GC-MS analysis was performed using GC-MS-5975C.

**Result:**

The RE showed potent antimicrobial activity against various ATCC cultures. The rose extract strongly inhibits the growth of ESBL resistant organism along with inhibition of biofilm formation in the ESBL resistant organism. The extract caused apoptotic and necrotic cell death in lung cancer cells. GC-MS analysis demonstrated the presence of several biologically active compounds such as Clindamycin, Phytol, Octanoic acid, and Stigmasterol which might be the reason for the therapeutic properties of the plant.

**Conclusion:**

This study shows the antimicrobial and biofilm inhibition activity against the clinical isolates of *Klebsiella pneumonia*. The study shows the cytotoxic and apoptotic activity in A549 cancer cell line. Thus, the plant may act as a potent antimicrobial drug against resistant strains.

**Supplementary Information:**

The online version contains supplementary material available at 10.1186/s12906-023-04222-2.

## Background

Herbal plants are a promising source of drug targets for various acute and chronic diseases. In developing nations, 60–90% of the population relies on herbal plants because of their efficacy and fewer side effects [1]. Several modern pharmaceuticals are made from herbs and medicinal plants based on the properties of the traditional knowledge of the plant. Understanding the pharmacological activity and bioactive components present in the plant further strengthens the traditional use of plants [2, 3]. In the traditional system of medicine, a single plant is utilized in multiple formulations to treat a variety of ailments. Some plants exhibit therapeutic characteristics in only a few parts due to the distribution of secondary metabolites, while the remaining parts of the plant may be devoid of any such properties. Therefore, it is crucial to screen both the whole plant and individual components to determine its medicinal value. The Chinese rose (*Rosa chinensis*) from the Rosaceae family is not only an ornamental plant but is rich in resources and widely used as food, spices and essential oil [4]. The important phytochemicals in the *R. chinensis* flowers are flavonoids such as Kaempferol, Quercetin, Apigenin etc. [5, 6]. The ethanolic extract of the flower showed anti-microbial activity against *Staphylococcus aureus* and *Klebsiella pneumonia.* The extract also showed antioxidant and anti-ageing properties. As a result, *R. chinensis* is a good candidate to examine for in vitro anti-bacterial and anti-proliferative properties.

The discovery of novel antimicrobial drugs is sparked by the emergence of various antibiotic resistance strains. The development of antibiotic resistance in gram negative bacteria becomes the key challenge in treating disease and lowering patient mortality and morbidity [7]. Identifying novel antimicrobial agents from plant-based compounds may result in anti-microbial drugs effective against the resistant strains. Plants contain various active compounds like polyphenols, coumarins, alkaloids, terpenoids and peptides that possess antimicrobial activity [2]. The antimicrobial activity of the plant extract is based on both the elimination and inhibition of pathogenic processes by the microorganisms [8].

Cancer is a multifaceted disease characterized by uncontrollable proliferation of cells to form tumors or neoplasms. Tumors can become hazardous and even fatal when they spread to the cancer through a process known as metastasis. The World Health Organization reported 10.3 million cancer deaths and 19.3 million new cases worldwide in 2020 [9]. In India, the National Cancer Registry Programme reported 14, 61,427 incident cancer cases for the year 2022 [10]. Breast, lung, and colon/rectum cancers were the most common types of cancer, while lung and breast cancers were the most prevalent in men and women respectively.

According to estimates, there will be 12.8% more cancer cases in 2025 than there were in 2020 [11]. Currently, different strategies like radiotherapy, chemotherapy, and surgery are used to treat cancer, either alone or in combination. Cancer chemotherapy utilizes drugs that act by destroying the mitotic spindle (taxol, vinblastine, and vincristine), blocking DNA synthesis (fluorouracil, mercaptopurine, and methotrexate), damaging the DNA (cisplatin, doxorubicin, and etoposide) and using inhibitor therapies (histone deacetylase, mechanistic target of rapamycin, poly (adenosine di-phosphate-ribose) polymerase [12]. However, chemotherapy has several detrimental side effects, due to which efforts have been concentrated on finding new molecular targets that would allow for minimal side effects. Therefore, drugs derived from plants used in traditional medicine are interesting possibilities in the hunt for new agents in cancer chemotherapies. In this study, we report the anti-microbial and anti-cancer properties of *R. chinensis*.

## Materials and methods

### Plant material

Rose flowers were procured from the flower bazaar at Koyambedu market in the month of April. The purchased flowers were authenticated by a research officer, at Siddha Central Research Institute. The flowers were checked for the presence of any foreign materials and the damaged flowers were eliminated due to the transportation process; the petals were checked for pest damage. In freshly chosen flowers the petals were removed separately and washed in clean tap water and distilled water. After washing, the flower was shade dried and ground into a coarse powder.

### Materials

Dulbecco’s modified Eagle medium (DMEM), Phosphate Buffered Saline (PBS), 3-(4,5- dimethylthiazol-2-yl)-2,5-diphenyltetrazolium bromide (MTT), foetal bovine serum (FBS), antibiotic-antimycotic solution, and dimethyl sulfoxide (DMSO) were purchased from HiMedia Laboratories, India. Acridine orange, ethidium bromide and Hoechst 33,342 were obtained from Sigma (St. Louis, USA). All reagents and chemicals used were of tissue culture and molecular biology grade.

### DNA isolation from rose flower petals

200 mg of flower petals were ground using a mortar and pestle in 500 µl of CTAB buffer (2% hexadecyltrimethylammonium bromide, 1.4 M NaCl, 0.2% β2-Mercaptoehanol, 20mM EDTA, 100mM Tris-HCl, pH 8) to isolate DNA. The contents are transferred into a microfuge tube and incubated at 55 °C for 30 min. Following the incubation period, the tubes were spun at 10,000 g for 10 min and the supernatant was collected separately in fresh tubes. To the supernatant 250 µl of chloroform: isoamyl alcohol (24:1) was added and the solution was mixed by inversion. The solution is to be centrifuged at 10,000 rpm for 10 min and the upper aqueous phase is carefully transferred to new microfuge tubes. 50 µl of 7.5 M ammonium acetate was added followed by 500 µl of ice-cold absolute ethanol and the tubes were inverted to precipitate DNA. The tubes were centrifuged at 10,000 rpm for 10 min and the pellet was completely air dried. The DNA was dissolved in sterile TBE (pH 8.0) water [13].

### Molecular identification of the plant

*rbcL-BF5’ATGTCACCACAAACAGAAAC3’* and *rbcL-724R 5’TCGCATGTACCTGCAGTAGC3’* primers were used for plant sample RBCL gene amplification. After the initial denaturation process at 95 °C for 5 min, the following thermal cycler (Applied Biosystems) profile was set to 35 cycles as follows: denaturation at 95 °C for 1 min; annealing at 54 °C for 45 s; and extension at 72 °C for 45 s. The final extension for the sample was maintained at 72 °C for 5 min. The amplified PCR products were electrophoresed on 1.5% agarose gel stained with 0.5 µg/mL ethidium bromide in TAE buffer (1x) to check for the presence or absence of bands. Gel imaging was performed using gel doc imaging system. The band size of amplified products was determined using 100 bp ladder. Using the BLAST program, the sequenced PCR product was aligned.

### Extraction of flower

100gm of the coarsely powdered flowers were mixed with 500 ml of acetone and the extraction was carried out by the maceration method for 48 h with frequent agitation at 120 RPM. Then the extract was filtered and concentrated using rotary evaporator (45 °C). The dried rose extract was preserved at -20℃ [14].

### Anti-microbial activity

#### Procuring the microbial culture

A total of 6 ATCC microorganisms were used in this study. *Enterobacter spp, Escherichia coli, Pseudomonas aeruginosa, Klebsiella pneumonia, Enterococcus faecalis and Staphylococcus aureus* were procured and maintained in the laboratory. ESBL resistant strains of *K.pneumonia* were procured from the central laboratory of Sree Balaji Medical College and Hospital after obtaining ethical clearance (001/SBMCH/IHEC/2021/1173).

#### Anti-microbial activity

The agar well diffusion method was employed to examine the anti-bacterial activity. The bacteria were infused into the nutritional broth and cultured overnight at 37℃. Following incubation, the turbidity of the organism was adjusted to 0.5 McFarland standards yielding a final inoculum of 1.5 × 10^8^ CFU/ml. Muller Hilton agar plates were lawn cultured by boring 6 mm of wells in the media. Each well was filled with different concentrations of the RE and the central well contained the standard drug. The plates were incubated for 24 h at 37℃ to allow the extract to diffuse. The zone of inhibition was measured in millimeters following the incubation period.

#### Biofilm inhibition assay

To perform the anti-biofilm activity, 96 microwell plates were used. In each well, 100 µl of the Muller Hilton broth containing the test organism in the stationary phase (OD = 0.6) was added. Each well received 100 µl of the extract at a different concentration. The plates were incubated for 24 h at 37℃. After incubation, the media was removed, and the plates were gently washed with distilled water. The plates were air dried after being fixed with 99% methanol. After a few min, 200 µl crystal violet (0.2%) was added. After 20 min, the excess crystal violet stain was removed from the plates by washing them in distilled water. The stain was eluted with 33% acetic acid and the biofilm growth was monitored by measuring OD at 570 nm using microplate reader [10]. The percentage of inhibition of biofilm by the extract against each resistant isolates was calculated using the formula: 100- {(OD of the treated wells/OD of control) *100} [15].

### Anti-proliferative activity against Lung cancer cell line

#### Cell culture

The National Centre for Cell Sciences (NCCS) (Pune, India) provided the human lung adenocarcinoma cell line, A549, which was maintained in 75cm^2^ tissue culture flasks with DMEM supplemented with 10% (v/v) FBS, 100 units/ml penicillin, and 100 g/ml streptomycin. For growth, the cells were kept in an environment of humidified 5% CO_2_ at 37 °C. 3T3-L1 the normal fibroblast cell line was obtained from NCCS and maintained in the same medium and same condition as used in A549 cell line.

#### Cell viability assay

The A549 and 3T3-L1 cell lines were cultivated in a 96-well plate with a density of 5 × 10^3^ cells/well. Following 24 h, they were treated with various concentrations of RE (100 to1000 µg/ml) for a treatment period of 48 h. The plates were incubated at 37 °C in a humidified atmosphere containing 5% CO_2_ in air. Following incubation, 100 µl MTT (5 mg/ml) dissolved in phosphate buffered saline (PBS) was added to each well for 4 h. The formazan crystals were dissolved in 100 µl DMSO and the color developed was measured at 540 nm using a microplate reader (Multiskan FC Microplate Photometer, Thermo Scientific). Cell viability was calculated as a percentage ratio and compared to untreated control (100%). The experiment was repeated in triplicates, and 50% inhibitory concentration was calculated as IC_50_ based on the reduced absorbance [16].

### Acridine orange/ethidium bromide staining (AO/EB)

A549 cells (1 × 10^5^) were plated in 24-well plate and treated with RE at IC_50_ concentration and left to incubate for 48 h. Following incubation, the cells underwent two PBS washes before being stained with equal parts of 10 µl of dye mixture (10 mg/ml AO and 10 mg/ml EB). The cells were examined under a fluorescence microscope at 200x magnification with an excitation filter of 480 nm [17].

### Hoechst 33,342 staining for nuclear apoptosis

In 6-well plates containing A549 cells (1 × 10^5^), RE at its IC_50_ concentration was treated for 48 h. The cells were then rinsed in PBS, fixed for 20 min in 4% paraformaldehyde, and stained for 20 min in the dark at 37 °C with Hoechst 33,342 (10 µg/ml). Excess stain was removed by washing with methanol and PBS and viewed under the fluorescence microscope at 200x magnification [18].

### GC-MS analysis of the rose extract

GC-MS analysis was performed using GC-MS-5975 C (AGILENT) to investigate the chemical presence in RE extract. 2 µl of the sample was injected and helium was used as the carrier gas. The samples were injected at the constant flow rate of 1.51 ml/min. The temperature of the column was maintained at 70℃. The run time for the GC-MS analysis was 30 min. The organic compounds were identified using the inbuilt mass spectra library of National Institute of Science and Technology (NIST-11).

### Statistical analysis

All the experiments were performed in triplicates. For the anticancer activity, data were represented as mean ± SEM, performed in triplicate (n = 9). Data was analysed using one-way ANOVA followed by Dunnett’s multiple comparison t-test). For the antimicrobial activity, data were represented as mean ± S.D, performed in triplicate (n = 3).

## Results

### Molecular identification of the plant

**Fig. 1:**
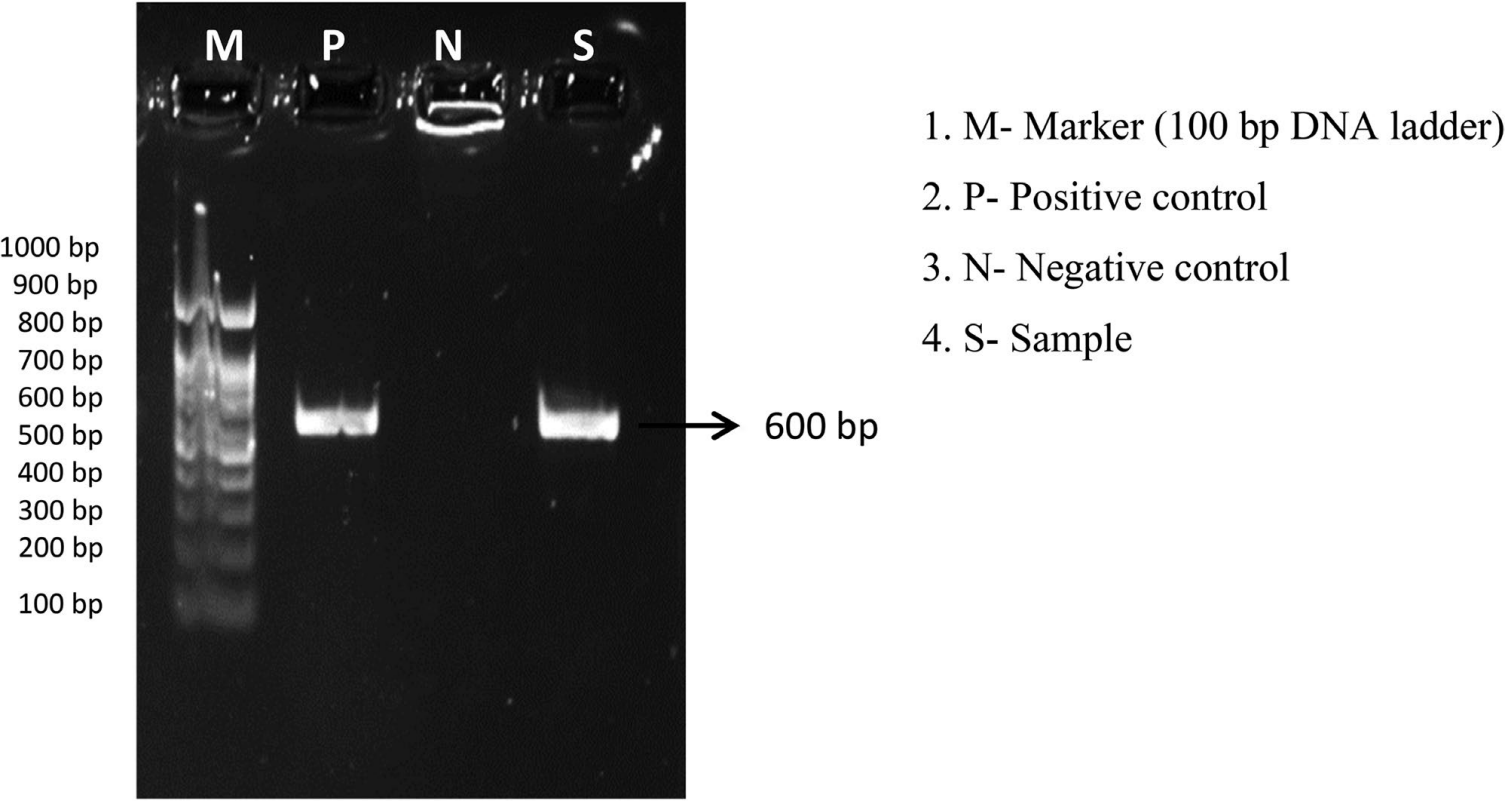
PCR product of the DNA isolated from the petals

**Fig. 2:**
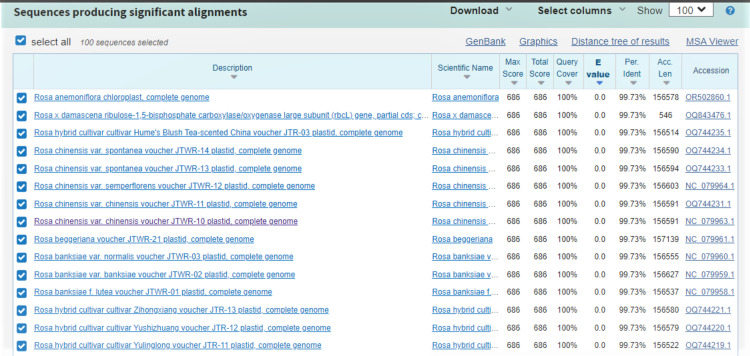
The BLAST analysis of the obtained sequence from the PCR product

DNA barcoding technique was used to identify the species of the plant. The sequence obtained using Sanger Sequencing method was analyzed using BLASTN. The result showed the sample was *R. chinensis* (Figs. 1 and 2).

### Anti-microbial activity of the RE

The agar well diffusion method was used to study the anti-microbial activity of diverse microorganisms. Maximum action against *S. aureus* was observed in the zone of inhibition, which was then followed by *P. aeruginosa, E. coli, Proteus, E. faecalis, and Enterobacter* (Table 1). Among the 4 ESBL resistant isolates, RE showed potent anti-microbial activity against the R1 strain and for the other three strains, it showed moderate activity (Table 2).

**Table 1:** Anti-microbial activity of RE against different ATCC cultures. Results were expressed in Mean ± SD

S. No	Organism name	Zone of Inhibition (mm)			
		25 µg	50 µg	75 µg	100 µg
1	*S. aureus*	7.15 ± 0.81	7.5 ± 0.063	8.3 ± 0.45	11.2 ± 0.81
2	*P. aeruginosa*	6.5 ± 0.09	6.8 ± 0.072	7.2 ± 0.16	10.5 ± 4.5
3	*E. coli*	6.2 ± 0.081	7.2 ± 0.9	8.1 ± 0.27	10.0 ± 1.8
*4*	*Proteus*	6.1 ± 0.063	7.3 ± 0.72	*8.5* ± 0.72	*9.7* ± 0.9
*5*	*E. faecalis*	7.1 ± 0.072	7.5 ± 0.81	8.3 ± 0.81	8.9 ± 2.7
*6*	*Enterobacter*	6.3 ± 0.045	6.8 ± 0.081	7.1 ± 0.054	7.4 ± 0.009

**Table 2:** Anti-microbial activity of RE against different ESBL resistant *K.pneumonia* isolates using disc diffusion method. % of biofilm inhibition at 1000 µg/ml and IC_50_ value of biofilm inhibition of RE against various resistant strains of *K.pneumonia* isolates. Results were expressed in Mean ± SD

S.NO	Resistant Cultures	Zone of inhibition (mm)				% of inhibition of biofilm formation at 1000 µg/ml	IC_50_ Value in inhibiting the biofilm formation
		25 µg/ml	50 µg/ml	75 µg/ml	100 µg/ml		
1	R1	6.7 ± 0.63	7.1 ± 0.81	7.5 ± 0.09	8.2 ± 0.081	62.06 ± 0.45	26.5 ± 0.45 µg/ml
2	R2	3.5 ± 0.009	4.2 ± 0.9	5.3 ± 0.072	6.5 ± 0.009	48.38 ± 0.63	51.8 ± 0.63 µg/ml
3	R3	4.3 ± 0.045	5.4 ± 0.45	6.1 ± 0.45	6.8 ± 0.18	60.71 ± 0.63	37.89 ± 0.63 µg/ml
4	R4	4.5 ± 0.072	5.6 ± 0.081	6.2 ± 0.072	6.9 ± 0.018	40 ± 1.8	66.09 ± 1.8 µg/ml

### Biofilm inhibition assay

**Fig. 3:**
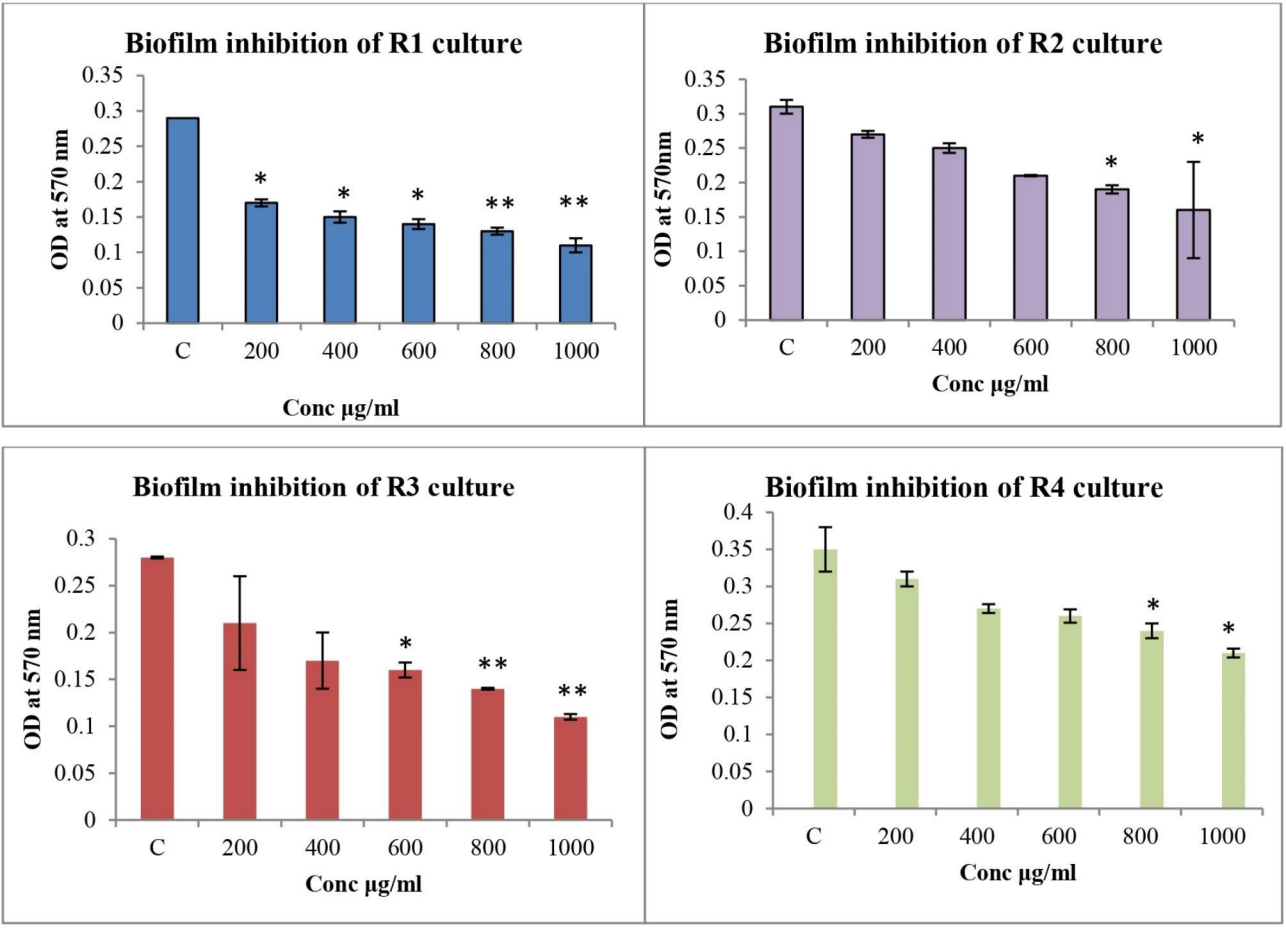
Graph shows the biofilm inhibition activity of the RE against the ESBL resistant strains of *K.pneumonia. *“C” refers to the control that shows the complete biofilm formation of the strains. The extract showed the dose dependent inhibition of biofilm formation. Results were expressed in Mean ± SEM. *p < 0.05 and **p < 0.01 for Control vs. 200, 400, 600, 800 and 1000 µg/ml. Data was analysed using one-way ANOVA followed by Dunnett’s multiple comparison t-test)

A biofilm inhibition assay was carried out for RE against the ESBL resistant isolates. The extract inhibited the biofilm formation for all the four isolates. Among the four isolates, biofilm inhibition activity of RE was highly observed in R3 isolates (Fig. 3).

### RE affects cell viability

**Fig. 4:**
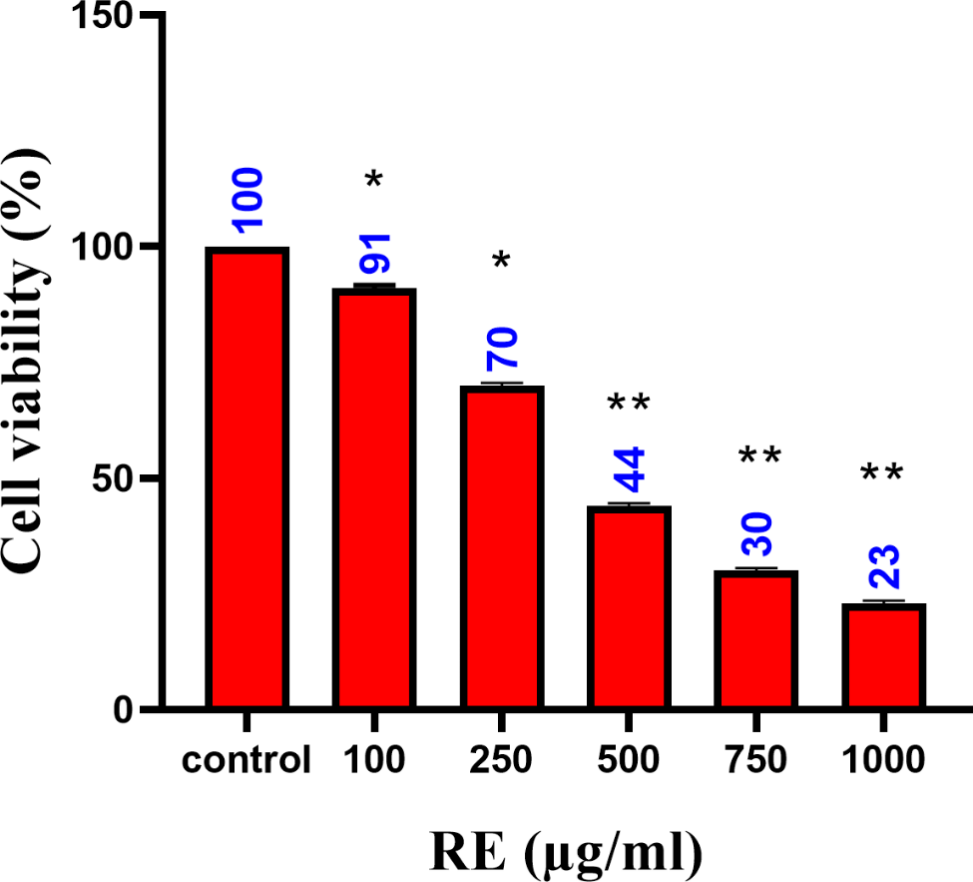
MTT assay. A549 cells were treated with different concentrations of rose extract for 48 h. Control is concentration 0 µg/ml of RE. Cell viability assay was performed using MTT and measuring absorbance at 48 h. Data were represented as mean ± SEM, performed in triplicate. *p < 0.05 and **p < 0.01 for 0 µg/ml vs. 100, 250, 500, 750 and 1000 µg/ml. Data was analysed using one-way ANOVA followed by Dunnett’s multiple comparison t-test)

The cells were treated with RE at various concentrations (100–1000 µg/ml) for 48 h to determine how RE affected the viability of A549 cells. MTT assay revealed a concentration-dependent effect with an incubation period of 48 h (Fig. 4). The IC_50_ of RE was found to be 430 µg/ml. In 3T3-L1 cell line, the cell viability was calculated, and the results showed 75.42% at the concentration of 1000 µg/ml (supplementary. Figure 1).

### Dual AO/EB staining

**Fig. 5:**
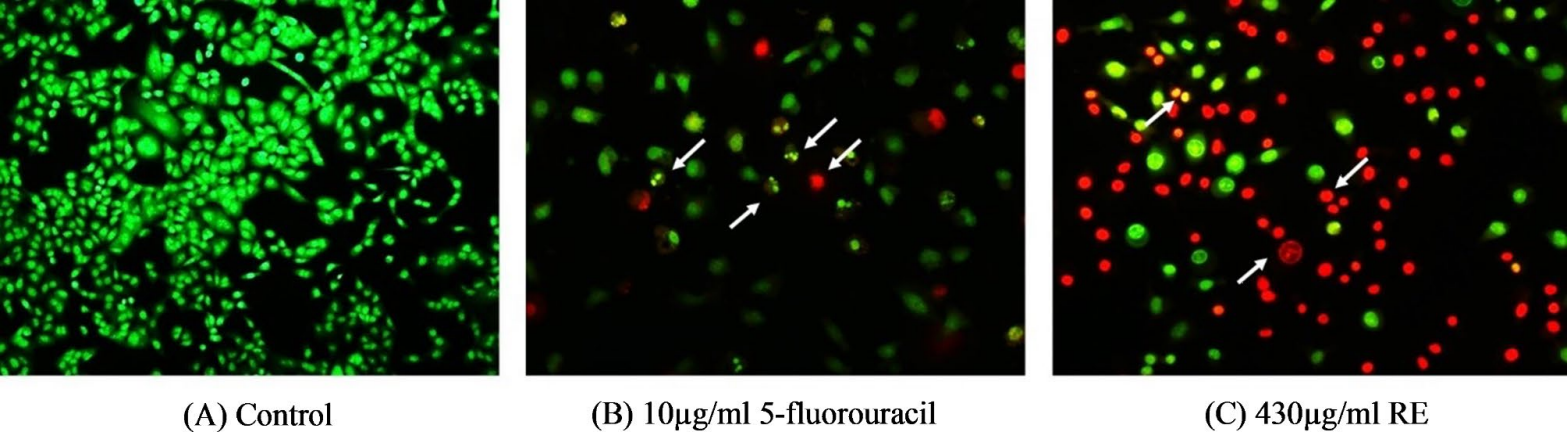
AO/EB staining. Fluorescence microscopy images of control, 5-fluorouracil (positive control) and RE-treated A549 cells. Apoptosis induction in cells was evaluated by AO/EB staining at 48 h. **(A)** Control (0 µg/ml); **(B)** 5-fluorouracil (10 µg/ml); and **(C)** IC_50_ value of RE (430 µg/ml). White arrows indicate apoptotic cells. Uniform green color – live cells; green with bright green dots – early apoptotic cells; orange – late apoptotic cells

The IC_50_ concentration of RE extract (430 µg/ml) and the positive control, 5-fluorouracil (10 µg/ml), were used to perform AO/EB staining on A549 cells (Fig. 5). Control cells showed uniform green color indicating live cells. The 5-fluorouracil treated A549 cells had vivid green spots in the nucleus, indicating nuclear fragmentation and chromatin condensation. In contrast, late apoptotic cells treated with 430 µg/ml of RE showed orange coloration with condensed and fragmented nuclei. Whereas cells treated with RE appeared orange with condensed and fragmented nuclei as they are late apoptotic cells. Necrotic cells show uniform orange stain with no condensed nuclei.

### Hoechst 33,342 staining

**Fig. 6:**
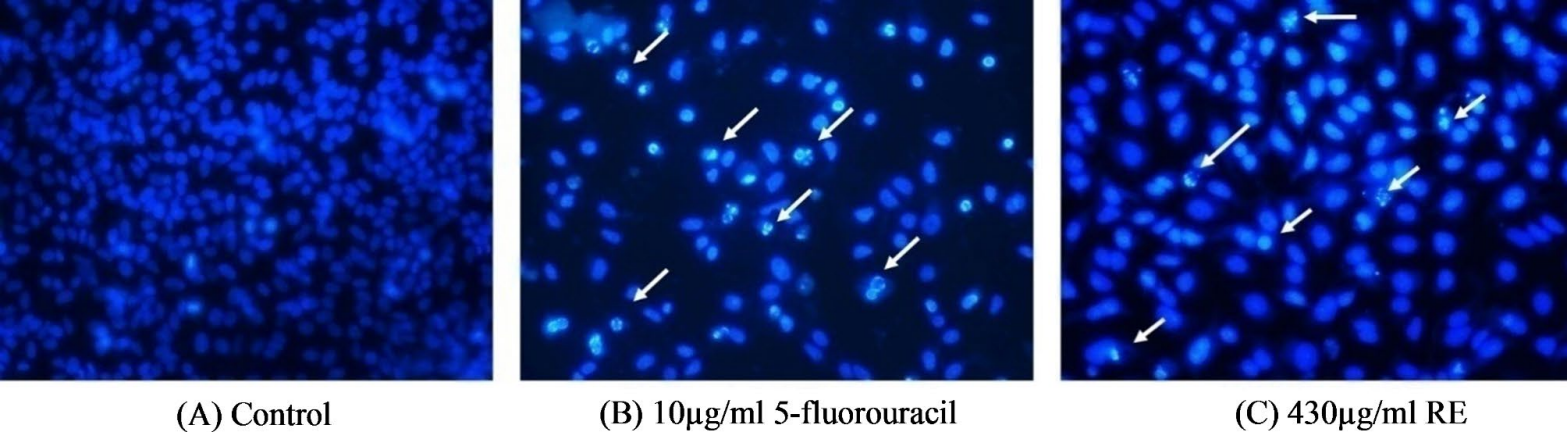
Hoechst staining. Fluorescence microscopy images of control, 5-fluorouracil (positive control) and RE-treated A549 cells. **(A)** Control (0 µg/ml); **(B)** 5-fluorouracil (10 µg/ml); and **(C)** IC_50_ value of RE (430 µg/ml). White arrows indicate condensed nuclei and apoptotic cells

Induction of apoptosis by RE on A549 cells was further confirmed using the Hoechst 33,342 stain (Fig. 6). The control cells exhibited dull blue fluorescence nuclei with round typical and orderly morphology. Contrarily, 5-fluorouracil (10 µg/ml), a positive control, and treatment with RE at 430 µg/ml led to the emission of vivid blue apoptotic nuclei as evident by contracted and disunited nuclei and chromatin at the nuclear membrane boundary.

### GC-MS analysis

**Fig. 7:**
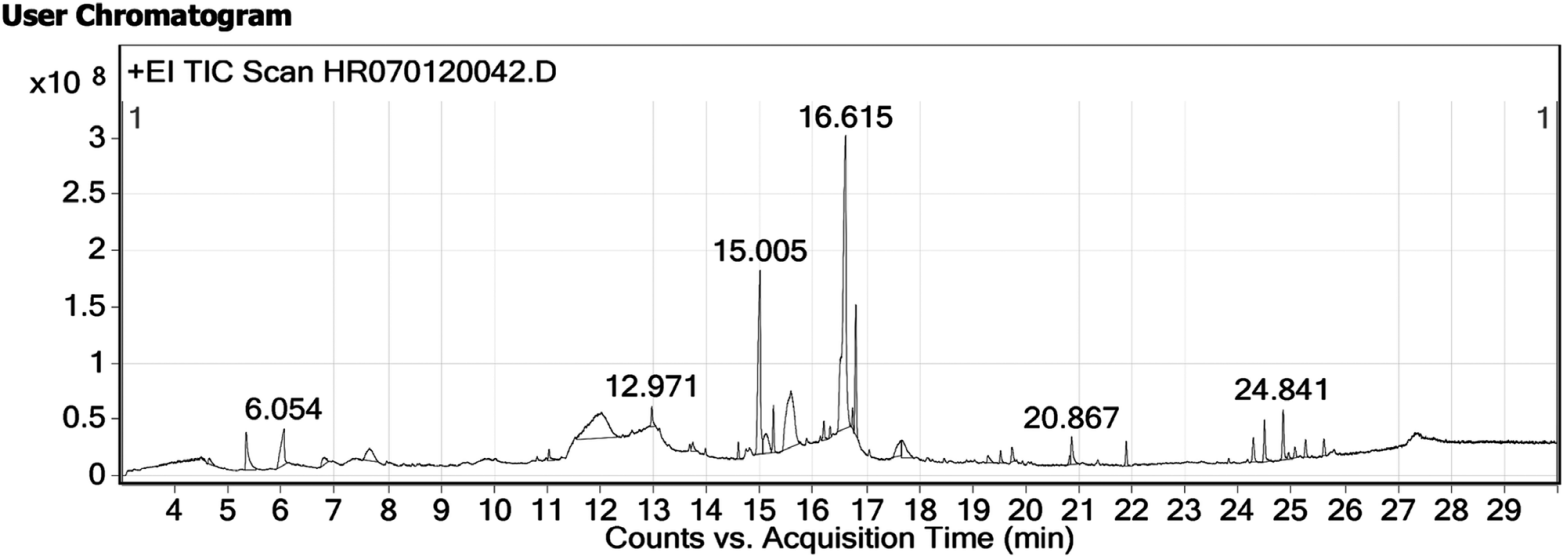
GC-MS chromatogram of the RE

GC-MS analysis of the RE revealed the presence of various compounds (Fig. 7). A total of 30 compounds were present in the RE. Most of the compounds like clindamycin, phytol, octanoic acid, and stigmasterol possess various biological activities (Table 3).

**Table 3:** List of compounds present in the RE

S.No	Compound Name	Retention time	Area (%)	Mass
1	Clindamaycin	4.65	0.49	424.2
2	Trans-3-Methyl-2-n-propylthiophane	5.34	3.09	144.1
3	Octanoic acid	6.05	3.78	144.1
4	Decanoic acid, 3-hydroxy-, methyl ester	6.82	0.86	202.2
5	1-Piperidinethiocarboxamide	7.66	2.40	144.1
6	Diethyl Phthalate	11.04	0.55	222.1
7	1,2,3,5-Cyclohexanetetrol, (1.alpha.,2.beta.,3.alpha.,5.beta.)-	12.03	13.43	148.1
8	Tetradecanoic acid	12.97	0.87	228.2
9	1-Ethynyl-3,trans(1,1-dimethylethyl)-4,cis-methoxycyclohexan-1-ol	13.74	0.56	210.2
10	Hexadecanoic acid, methyl ester	14.60	0.54	270.3
11	n-Hexadecanoic acid	15.01	12.71	256.2
12	(E,Z,Z)-2,4,7-Tridecatrienal	15.12	2.91	192.2
13	2(3 H)-Naphthalenone, 4,4a,5,6-tetrahydro-	15.59	12.39	148.1
14	11-Octadecenoic acid, methyl ester	16.21	0.80	296.3
15	Phytol	16.32	0.37	296.3
16	9-Octadecenoic acid, (E)-	16.62	28.69	282.3
17	9,12-Octadecadienoic acid (Z,Z)-, methyl ester	16.74	0.57	294.3
18	Tetracyclo[6.3.2.0(2,5)0.0(1,8)]tridecan-9-ol, 4,4-dimethyl-	17.65	1.96	220.2
19	Cis-5,8,11,14,17-Eicosapentaenoic acid	17.68	2.37	302.2
20	Ferrocene, 1,1’-diacetyl-	19.29	0.57	270
21	Hexadecanoic acid, 2-hydroxy-1-(hydroxymethyl)ethyl ester	19.53	0.55	330.3
22	Dibenz[a,c]cyclohexane, 2,4,7-trimethoxy-	19.74	0.70	284.1
23	Butyl 9,12-octadecadienoate	20.82	0.34	336.3
24	6-Octadecenoic acid	20.87	1.67	282.3
25	Campesterol	24.29	1.18	400.4
26	Stigmasterol	24.50	1.89	412.4
27	beta.-Sitosterol	24.84	2.19	414.4
28	Ursodeoxycholic acid	24.95	0.29	392.3
29	Cholest-4-en-3-one	25.06	0.51	384.3
30	4,22-Stigmastadiene-3-one	25.27	0.76	410.4

## Discussion

The increasing resistance of microorganisms to various antibiotics necessitates the development of effective new anti-microbial agents. Researchers and pharmaceutical companies screen for biological activity in natural products to look for secondary metabolites effective against various infections caused by bacteria [19]. *R. chinensis* commonly considered the queen of flowers was chosen to evaluate its anti-bacterial activity against various pathogens. Despite its ornamental purpose it also possesses some medicinal properties including anti-oxidant activity [20]. To confirm the plant species, DNA barcoding was used, which sequences the standard region of DNA, which helps to elucidate the identification of species. DNA barcoding confirms the flower belongs to *R. chinensis.*

The standard technique used to assess the antibacterial activity is the qualitative approach known as the well diffusion method [21]. The anti-bacterial activity of flower extract against various gram positive, gram negative as well as ESBL resistant isolates were examined. When compared to other gram-negative bacteria in all ATCC cultures, RE was efficient against *S. aureus*. Previous studies also demonstrated that plant extracts showed more activity in gram positive bacteria when compared to gram negative bacteria [22, 23]. The compound from the ethanolic extract of *R. chinensis* showed good anti-bacterial activity against *S. aureus* with a MiC_50_ value of 8.51 ± 0.26 µg/mL [5]. In the present study, acetone extract was chosen to study the bioactivity of the flower and the identification of the compounds present in the flowers of *R. chinensis.* Few studies showed acetone is the best solvent for extracting anti-oxidant and anti-microbial properties, considering this, the present study used acetone for the extraction purpose [24–26].

In the present study, ESBL resistant *K. pneumonia* was chosen to evaluate the anti-microbial activity. ESBL resistant organisms show resistance to various antibiotics including all penicillin, and cephalosporin like combinations of clavulanic acid and monobactams. The prevalence of nosocomial infections caused by ESBL *K. pneumoni*a is rising worldwide, and there are few effective treatments available [27]. RE extract showed a good zone of inhibition against all ESBL resistant organisms. The formation of biofilm by bacteria poses a serious threat to medicine because of the increased use of medical implants. Inhibiting biofilm formation is very important because inside the biofilm a greater number of microorganisms are present and show increased resistance to antibiotics [28]. The prevention of biofilm formation in all the isolates by RE extract further proves the efficacy of the anti-microbial activity. RE showed more than 60% of biofilm inhibition for two resistant isolates. Previous studies also showed that the acetone extract of *J. gossypiifolia* showed good biofilm inhibition activity when compared to other extracts [29]. Thus, inhibiting biofilm formation against resistant organisms is a very important anti-microbial property. The anti-bacterial action of the extract may be increased by the active substances found in the plant, such as phytol and clindamycin. The anti-bacterial action needs to be explored further through research.

The use of anti-cancer compounds derived from plants as novel treatment agents for cancer is not new. Probably the most well-known is Paclitaxel (Taxol®), a taxane diterpene found in the barks of *Taxus brevifolia* Nutt. (Western yew) [30]. Following this, other plant compounds have been identified that have important anti-cancer effects, including activation of the DNA repair mechanism and/or stimulation of the cellular anti-oxidant enzyme system. These plant chemicals are classified as primary and secondary metabolites, including alkaloids, flavonoids, lignans, saponins, terpenes, taxanes, vitamins, minerals, glycosides, gums, oils, and biomolecules [29, 31–35]. The edible flower *R. chinensis* has been evaluated to contain phytochemicals like catechin, hesperidin, naringenin, procyanidins (A2, B1 and B2), and anthocyanin [36]. In the present study, RE was evaluated for its anti-cancer effect on the lung cancer cells, A549. We showed that the RE had a concentration-dependent effect on A549 cells and recorded an IC_50_ at 430 µg/ml. Previous studies showed that ethyl acetate extract showed a good growth inhibition effect on lung cancer cell line at the concentration of 152.3 µg/ml [37]. The difference in activity might be due to the different solvent extraction process. Necrosis and apoptosis are the two basic processes by which cells undergo programmed death. Necrosis occurs when a cell loses its metabolic or membrane integrity whereas, apoptosis happens when the intracellular death program is activated [38].

Apoptosis is the major programmed cell death that displays many alterations in cellular biochemistry and morphology. The effects of apoptosis include cell shrinkage, chromatin condensation, intranucleosomal DNA fragmentation, cytoplasmic vacuolization, and membrane blebbing with shedding of apoptotic particles [39]. EB penetrates cells with damaged membranes, such as late apoptotic and dead cells, displaying orange-red fluorescence attaching to concentrated DNA fragments or apoptotic entities, whereas AO penetrates intact membranes in normal and early apoptotic cells, fluorescing green when coupled to DNA [40]. The presence of nuclear condensation and DNA fragmentation, which are signs of early and late apoptotic bodies, showed that RE caused apoptosis in A549 cells. Furthermore, DNA-binding dyes like Hoechst 33,342 can be used to observe nuclear condensation. The apoptosis rate of the RE treatment group is higher than the control group, as seen by the decreased number of cells that exhibit blue fluorescence from Hoechst 33,342. The induction of apoptosis is considered a promising method for treating cancer. Hence, detecting tumour cell apoptosis is more crucial than determining the survival of the tumour cells.

Clindamycin is a known antibiotic that effectively works against *Chlamydia trachomatis* and certain protozoa [41]. Stigmasterol found in many plants, possesses anti-pyretic and anti-neoplastic activity [42]. Octanoic acid is a long chain fatty acid and possesses anti-candida activity [43]. Phytol is a diterpene which displays various biological activities including anti-microbial, immunomodulating, anti-oxidant, anti-inflammatory and anti-cancer activities [44]. Thus, the study shows potent anti-microbial activity against the ATCC cultures and ESBL resistant *K. pneumonia*. The study also showed anti-cancer activity against the lung cancer cell line. This preliminary screening study showed the pharmacological activity of *R. chinensis*. Further study must be conducted to show the mechanistic approach of the plant.

## Conclusions

The study shows the anti-microbial and biofilm inhibition activity against the clinical isolates of *K. pneumonia*. The study shows the cytotoxic and apoptotic activity in A549 cancer cell line. Thus, the plant may act as a potent anti-microbial drug against resistant strain. Further study must be performed to further investigate the therapeutic approach of the plant.

### Electronic supplementary material

Below is the link to the electronic supplementary material.

Supplementary Material 1

## Data Availability

The datasets used and/or analyzed during the current study are available from the corresponding author on reasonable request.

## Abbreviations

RE: Rose extract
GC-MS: Gas chromatography Mass Spectrometry
AO/EB: Acridine Orange/Ethidium Bromide
