# Supplementary material for: Anti-microbial and anti-cancer efficacy of acetone extract of Rosa chinensis against resistant strain and lung cancer cell line
Source: BMC Complement Med Ther. 2023 Nov 10;23:406. doi: 10.1186/s12906-023-04222-2 (PMC10636979; doi:10.1186/s12906-023-04222-2)
Supplement: Supplementary file 1 — Supplementary Material 1 [file 12906_2023_4222_MOESM1_ESM.docx]

**Supplementary figure.1**

**Fig. S1. MTT assay.** 3T3-L1 cells were treated with different concentration of rose extract (RE) for 48h. Control is concentration 0µg/ml of RE. Cell viability assay was performed using MTT and measuring absorbance at 48h. Data were represented as mean±SEM, performed in triplicate. **p*<0.05 vs. 100, 250, 500, 750 and 1000µg/ml. Data was analysed using one-way ANOVA followed by Dunnett's multiple comparison t-test).

**>Rosa chinensis (99.15%) Sequence**

TTTTTTTCACCCACAAACAGAGACTAAAGCAAGTGTTGGATTCAAAGCTGGTGTTAAAGA

TTATAAATTGACTTATTATACTCCGGAGTATGAAACCAAAGATACTGATATCTTGGCAGC

ATTTCGAGTAACTCCTCAACCTGGAGTTCCGCCTGAGGAAGCAGGGGCAGCGGTAGCTGC

GGAATCTTCTACTGGTACATGGACAACTGTATGGACTGATGGGCTTACCAGTCTTGATCG

TTACAAAGGGCGATGCTACCACATTGAACCTGTTGCTGGAGAAGAAAGTCAATTTATTGC

TTATGTAGCTTACCCCTTAGACCTTTTTGAAGAGGGTTCGGTTACTAACATGTTTACTTC

CATCGTAGGTAATGTGTTTGGGTTCAAGGCCTTGCGCGCTCTACGTCTGGAGGATTTACG

AATCCCTACTGCTTATGTTAAAACTTTCCAAGGCCCGCCTCACGGGATCCAAGTTGAAAG

AGATAAATTGAACAAGTATGGCCGCCCCCTATTGGGATGTACTATTAAACCTACAATTGG

GGTGATCCGCTAAAAATTACGGTAGAGCAGTTTATGAATGTCTCCGCGGTGGACTTGGGA

TTTAAACAAA
